# Protection via a ROM4 DNA vaccine and peptide against *Toxoplasma gondii* in BALB/c mice

**DOI:** 10.1186/s12879-016-2104-z

**Published:** 2017-01-11

**Authors:** Yali Han, Aihua Zhou, Gang Lu, Guanghui Zhao, Lin Wang, Jingjing Guo, Pengxia Song, Jian Zhou, Huaiyu Zhou, Hua Cong, Shenyi He

**Affiliations:** 1 Department of Parasitology, Shandong University School of Medicine, Jinan, Shandong Province 250012 People’s Republic of China; 2 Department of Pediatrics, Provincial Hospital Affiliated to Shandong University, Shandong University School of Medicine, 250021 Jinan, Shandong Province People’s Republic of China; 3 Qilu Hospital of shandong University, Qingdao, 266035 Shandong Province People’s Republic of China; 4 Department of Ji Nan Children’s Hospital, 250022 Jinan, Shandong Province People’s Republic of China

**Keywords:** *Toxoplasma gondii*, Vaccine, ROM4, Peptide, Bioinformatics, Immunization strategy

## Abstract

**Background:**

*Toxoplasma gondii* (*T. gondii*) is an obligate intracellular protozoan parasite with a broad host range including most warm-blooded animals, including humans. *T. gondii* surface antigen 1 (SAG1) is a well-characterized *T. gondii* antigen. *T. gondii* expresses five nonmitochondrial rhomboid intramembrane proteases, TgROM1-5. TgROM4 is uniformly distributed on the surface of *T. gondii* and involved in regulating MIC2, MIC3, MIC6, and AMA1 during *T. gondii* invasion of host cells. Bioinformatics have predicted ROM4 B-cell and T-cell epitopes. Immunization strategy is also a key factor in determining the effectiveness of the immune response and has gained increasing attention in *T. gondii* vaccine research. In this study, we used a DNA prime-peptide boost vaccination regimen to assess the protective efficacy of various vaccination strategies using TgROM4.

**Methods:**

We identified a polypeptide (YALLGALIPYCVEYWKSIPR) using a bioinformatics approach, and immunized mice using a DNA-prime and polypeptide-boost regimen. BALB/c mice were randomly divided into six groups, including three experimental groups (peptide, pROM4 and pROM4/peptide) and three control groups (PBS, pEGFP-C1 and pSAG1). Mice were then immunized intramuscularly four times. After immunization, IgG and cytokine productions were determined using enzyme-linked immunosorbent assays. The survival time of mice was evaluated after challenge with tachyzoites of *T. gondii* RH strain. Additionally, the number of cysts in the brain was determined after intragastric challenge with cysts of *T. gondii* PRU strain.

**Results:**

Mice vaccinated with different immunization regimens (peptide, pROM4 and pROM4/peptide) elicited specific humoral and cellular responses, with high levels of IgG, IgG2a, and interferon (IFN)-γ. Moreover, IgG, IgG2a and IFN-γ levels were highest in the pROM4/peptide group. Immunized mice, especially those in the pROM4/peptide group, had prolonged survival times after challenge with tachyzoites and reduced numbers of brain cysts after infection compared with negative controls.

**Conclusion:**

A DNA prime-peptide boost regimen based on ROM4 elicited the highest level of humoral and cellular immune responses among immunization regimens, and may be a promising approach to increase the efficacy of DNA immunization.

## Background

*Toxoplasma gondii* (*T. gondii*) is an obligate intracellular protozoan parasite with an extremely broad host range inclusive of almost all warm-blooded animals, including human [1]. Toxoplasmosis is a zoonotic parasitic disease with a worldwide distribution that poses a serious risk to animal husbandry and human health [2]. *T. gondii* infection is usually asymptomatic, but not in immunosuppressed patients (e.g. organ transplantation, malignant tumor radiotherapy, and HIV infection). Infection can spread widely, involving the brain, eyes, lymph nodes, can damage multiple organs, and even cause death. *T. gondii* infection in pregnant women can be transmitted to the fetus via the placenta, leading to miscarriage, premature birth, stillbirth, fetal malformation, and eye toxoplasmosis [3].

There are three infectious stages in the *T. gondii* life cycle: tachyzoites, cysts, and oocysts [4]. Tachyzoites cause acute infection, while chronic infection is mainly mediated by bradyzoites in cysts. When the host immune function is compromised, bradyzoites transform to tachyzoites [3]. Current drugs are effective for the treatment of toxoplasmosis (acute or reactivated) but they cause many side effects, especially in the fetus, and have no effect on cysts and bradyzoites [5]. Therefore, a safe and effective vaccine is important for the prevention and control of toxoplasmosis. DNA vaccines induce a persistent and strongly protective immune response [6, 7]. For example, *T. gondii* surface antigen 1 (SAG1) induces effective and durable humoral and cellular immune responses in immunized mice and is regarded as a standard compared to other antigens [8].

*T. gondii* expresses several proteases, including serine, cysteine, and aspartic proteases, which play an important role in infection [9–11]. Rhomboid proteases belong to the serine protease family. *T. gondii* expresses 5 nonmitochondrial rhomboid intramembrane proteases, TgROM1 to TgROM5. ROM proteins affect the cleavage of microneme protein (MIC), which plays an essential role in *T. gondii* invasion [12, 13]. TgROM4 is expressed primarily in tachyzoites, at lower levels in bradyzoites, and is weakly detected in sporozoites [12, 13]. TgROM4 is localized to the plasma membrane of *T. gondii* and uniformly distributed on the parasite surface [12, 13]. When *T. gondii* effectively invades host cells, TgROM4 plays an important role in regulating microneme protein 2 (MIC2), MIC3, MIC6, and apical membrane antigen 1 (AMA1) [14–16]. Li et al. showed that a TgROM1 DNA vaccine was protective against *T. gondii* in mice [17]. We therefore used bioinformatics to examine whether ROM4 is antigenic. Bioinformatics is a powerful approach for genomics and proteomics and has been widely used to predict and analyze protein antigenic epitopes [18, 19].

Immunization strategy is also a key factor in determining the effectiveness of the immune response and has gained increasing importance in vaccine research [20, 21]. In particular, a prime-boost vaccination strategy induced a more efficient humoral and cellular immune response [22–24]. Studies have shown that a synthetic multiple antigenic peptides (MAP) vaccine induces protective immunity against intracellular parasites, including *T. gondii* [25, 26].

In the present study, we analyzed the ROM4 protein to identify potential antigenic epitopes using a bioinformatics approach. A eukaryotic expression plasmid pEGFP-C1-ROM4 (pROM4) was constructed and used in a DNA prime-peptide boost vaccination regimen to immunize mice and assess protection against *T. gondii* infection. Furthermore, we assessed the protective efficacy of different vaccination strategies (peptide, pROM4, and DNA/peptide).

## Methods

### Prediction of linear-B cell and Th cell epitopes

The TgROM4 (scaffold no. TGG995368, chromosome VIII) nucleotide (GenBank ID: AY704175.1) and amino acid sequences (GenBank ID: AAU11320.1) were obtained from GenBank. Protein epitopes determine antigen specificity [27, 28]. The linear-B cell epitopes of ROM4 were analyzed using DNASTAR (Madison, WI, USA). The PROTEAN subroutine was used to predict the antigenic index and ROM4 surface probability. Peptides with a good antigenic index and surface probability were chosen. The Immune Epitope Database (IEDB) (http://tools.immuneepitope.org/mhcii) online service was used to analyze the half-maximal inhibitory concentration (IC50) values of peptides that bind to the major histocompatibility complex (MHC) class II molecules of ROM4.

### Mice and parasites

Female BALB/c mice (7–8 weeks old) were purchased from the Shandong University Laboratory Animal Center (Shandong, China). All mice were maintained under specific-pathogen-free conditions. All animal experiments were approved by the Ethics Committee on Animal Experiments of the Medical School of Shandong University.

The *T. gondii* RH strain was used to challenge BALB/c mice. Tachyzoites were extracted from human foreskin fibroblast cells 1 h before injection to ensure freshness prior to challenge. About 8 × 10^9^ tachyzoites were used to create soluble tachyzoite antigens (STAg) after isolation by centrifugation, and resuspended in sterile PBS as previously described [5], while about 8 × 10^6^tachyzoites were used to extract total RNA with TRIzol Reagent (Life Technologies, Carlsbad, CA, USA). After tachyzoites were lysed with 1 ml TRIzol, 0.2 ml chloroform was added, and the homogenate was separated into three layers after centrifugation. RNA was precipitated from the upper aqueous layer with isopropanol and then was washed to remove impurities. RNA was resuspended in RNase-free water and incubated in a water bath at 55–60 °C for 10–15 min. The RNA was reverse-transcribed into cDNA with RevertAid First Strand cDNA Synthesis Kit according to the manufacturer’s protocol (Thermo Scientific, Carlsbad, CA, USA)

### Eukaryotic expression plasmid construction and preparation

The whole SAG1 open reading frame (ORF) was amplified by polymerase chain reaction (PCR) from *T. gondii* tachyzoite cDNA (forward primer: 5′-CCGCTCGAGCTATGTCGGTTTCGCTGCACCAC −3′, reverse primer: 5′-CGGAATTCTCACGCGACACAAGCTGCGAT-3′). *Xho*I and *Eco*RI restriction sites are underlined, respectively. The ROM4 ORF was amplified by PCR from *T. gondii* tachyzoite cDNA (forward primer: 5′-CCGCTCGAGTGGCGTCCCCTCACGGATCC-3′, reverse primer: 5′-GGGGTACCTTACGGTTCAAGGTAATACTGCGC-3′). *Xho*I and *Kpn*I restriction sites are underlined, respectively.

The SAG1 and ROM4 DNA fragments were respectively inserted into a pEASY-T1 vector (TransGen Biotech, Beijing, China). After sequencing, SAG1 and ROM4 were respectively subcloned into the eukaryotic expression plasmid pEGFP-C1 (Novagen, Billerica, MA, USA) to form pEGFP-C1-SAG1(pSAG1) and pEGFP-C1-ROM4 (pROM4), respectively using the *Xho*I, *Eco*RI and *Xho*I, *Kpn*I restriction sites. The new recombinant plasmids were then used to transfect HEK 293-T cells.

Recombinant pSAG1 and pROM4 were transformed into *Escherichia coli* DH5α. After being verified by PCR, double restriction enzyme digestion and double stranded sequencing, recombinant plasmids were extracted using an endotoxin-free mega kit following the manufacturer’s instructions (Qiagen, Hilden, Germany), and stored at −20 °C until use. pSAG1 and pROM4 concentrations were determined by A260/A280 measurement.

### Preparation of polypeptide

ROM4 peptide 405–424 (YALLGALIPYCVEYWKSIPR) was synthesized by SynPeptide Co Ltd (Shanghai, China), and purity was confirmed by analytic HPLC.

### Expression of pEGFP-C1-ROM4 in HEK 293-T cells

HEK 293-T cells were maintained in Dulbecco’s Modified Eagle Medium (DMEM) supplemented with streptomycin (100 mg/ml), penicillin (100 IU/ml) and 10% fetal bovine serum (FBS), at 37 °C in a humidified atmosphere with 5% CO2. Before transfection, HEK293 cells (about 1–2 × 10^5^/well) were transferred into Costar 6-well culture plates (Sigma-Aldrich, St. Louis, MO, USA). When HEK 293-T density reached 80–90%, pEGFP-C1, pSAG1 and pROM4 were transfected with Lipofectamine 2000 reagent (Invitrogen, Carlsbad, CA, USA) according to the manufacturer’s protocol. Plasmids (2.5 μg/well) were mixed with Lipofectamine 2000 reagent (7 μL/well) in DMEM, incubated at room temperature for 20 min, and then added drop by drop on to HEK 293-T cells. After incubation for 6 h, the medium was exchanged with medium containing 10% FBS. After 48 h, the cells from different groups (control, pEGFP-C1, pSAG1 and pROM4) were observed using fluorescence microscopy under a blue laser.

### Immunization and challenge

All BALB/c mice were divided randomly into six groups (27 per group). Mice were immunized twice at 2-week intervals with PBS (100 μl), pEGFP-C1 (100 μg), pSAG1 (100 μg), pROM4 (100 μg), peptide (100 μg), or pROM4 (100 μg)/peptide (100 μg) by intramuscular injection. All groups were immunized four times. The last group was injected with pROM4 the first two times and with peptide the last two times. 

Two weeks after the final immunization, seven mice per group were euthanized and splenocytes harvested under aseptic conditions for cytokine detection. Ten mice from each group were challenged intraperitoneally with *T. gondii* RH strain (1 × 10^4^ tachyzoites). Changes in health status were observed and survival times recorded. The remaining mice were infected intragastrically with 20 cysts of *T. gondii* PRU strain. At 1 month after challenge, brains were removed and homogenized in 1 ml PBS. The number of cysts in each brain was determined by counting three samples of 10 μl from the homogenate via an optical microscope, and the average value was used to evaluate the effect of the vaccine.

### Determination of antibodies

Serum samples were collected from all mice prior to each immunization and 2 weeks after the final injection. Anti-*T. gondii* IgG, IgG1, and IgG2a antibodies were detected using enzyme-linked immunosorbent assay (ELISA). Briefly, 96-well plates (Costar) were coated with STAg (10 μg/well) and incubated at 4 °C overnight. Plates were washed three times with special ELISA solution and blocked with PBS containing 1% BSA for 2 h at room temperature, followed by three washes. Plates were then incubated with PBS-diluted mouse sera for 1 h at 37 °C. After washing, plates were incubated with horseradish peroxidase (HRP)-conjugated anti-mouse IgG (diluted 1:4000 in PBS-1% BSA), IgG1 (1:2000), and IgG2a (1:2000) for 1 h at 37 °C, washed with ELISA solution, with orthophenylene diamine (Sigma) and 0.15% H_2_O_2_ added. Plates were then incubated in the dark for 30 min at 37 °C, and the reaction stopped by adding 2 M H_2_SO_4_. The optical density was measured at 490 nm using an ELX800 ELISA reader (BioTek, Winooski, VT, USA). All samples were run four times.

### Cytokine assays

Spleens were isolated from seven mice per group 2 weeks after the last immunization and used to form a cell suspension adjusted to 1 × 10^6^ cells/ml. Then, 1 × 10^5^ spleen cells was added to each well in a 96-well plate with 10 μl non-specific irritant Con A (5 μg/ml) and cultured at 37 °C in 5% CO2. Cell-free supernatants were harvested and assayed for interleukin-2 (IL-2) and IL-4 at 24 h, IL-10 at 72 h, and IL-12 (p70) and interferon (IFN)-γ at 96 h. The concentrations of cytokines were measured by ELISA according to the manufacturer’s instructions (R&D Systems, Minneapolis, MN, USA). The detection limits of the assays for IFN-γ, IL-2, IL-4, IL-10 and IL-12 (p70) were 2.1 pg/mL, 3.5 pg/mL, 2.3 pg/mL, 1.97 pg/mL and 2.24 pg/mL, respectively. All samples were run four times.

### Statistical analysis

SPSS 17.0 (IBM, Chicago, IL, USA) was used for statistical analysis. The mean of total IgG, IgG1, IgG2a, cytokine levels and cyst numbers among the different groups were analyzed and determined by one-way analysis of variance (ANOVA). Mouse survival time was compared using the Kaplan-Meier method. When a significant difference (*P* < 0.05) was observed among treatments, a Tukey’s studentized range test was used for post-test comparisons.

## Results

### Epitope analysis

**Fig. 1:**
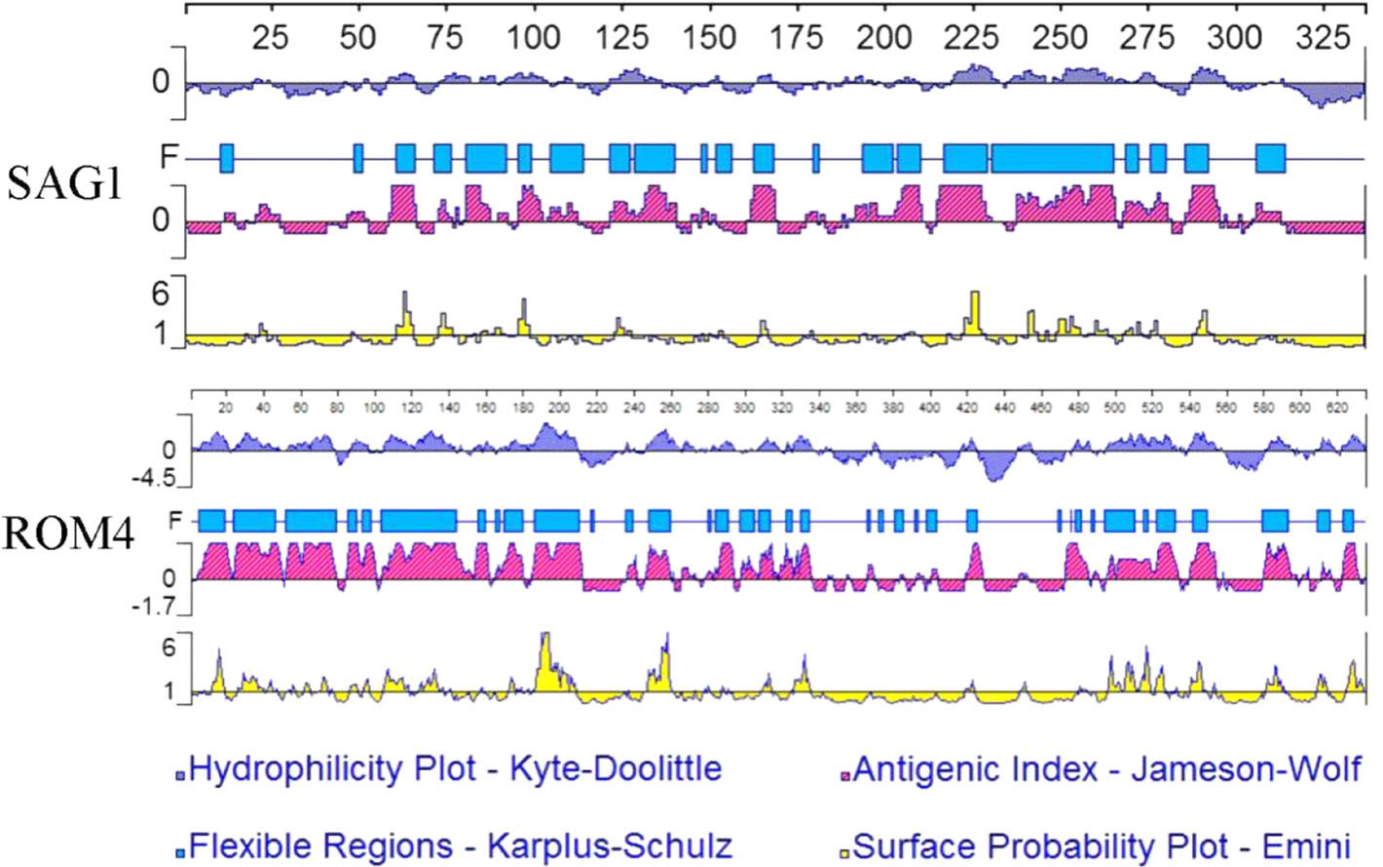
The linear-B cell epitopes of SAG1 and ROM4 predicted by DNASTAR in antigenic index, hydrophilicity plot, flexible regions and surface probability rules

DNASTAR was used to determine hydrophilicity plots, flexible regions, antigenic index, and surface probability. SAG1 had an excellent antigenic index and surface probability, making it a good vaccine candidate (Fig. 1). Prediction results indicated that ROM4 had a better antigenic index than SAG1. Moreover, ROM4 had significantly more surface probability and more flexible regions than SAG1.

The ROM4 Th predicted epitopes, and the IC50 values of ROM4 peptides binding to MHC class II molecules, and the minimum percentile ranks of each ROM4 MHC II allele are listed in Table 1. The minimum percentile ranks of HLA-DRB1*01:01, H2-IAb, H2-IAd, and H2-IEd alleles of ROM4 were smaller than those of SAG1, indicating that ROM4 may have better Th epitopes than SAG1.

**Table 1:** IC50 values for SAG1 and ROM4 peptide binding to MHC class II molecules obtained using the immune epitope database^a^

MHC II Allele^b^	Start-Stop^c^		Percentile Rank^d^	
	SAG1	ROM4	SAG1	ROM4
HLA-DRB1*01:01	12–26	570–584	0.88	0.09
	35–49	399–413	2.74	0.6
H2-IAb	26–40	483–497	2.15	1.75
	297–313	42–56	2.81	1.95
H2-IAd	21–35	502–516	0.34	0.39
	168–182	529–543	1.22	0.58
H2-IEd	14–28	179–193	18.45	4.91
	34–48	412–426	30.62	11.21

^a^ The immune epitope database (http://tools.immuneepitope.org/mhcii). The prediction was run for three times

^b^ H2-IAb, H2-IAd and H2-IEd alleles are mouse MHC class II molecules; the HLA-DRB1*01:01 allele is a human MHC class II molecule

^c^ We chose 15 amino acids for analysis each time

^d^ Low percentile = high level binding, high percentile = low level binding

### Identification and expression of recombinant plasmid

In pSAG1-, pROM4- and vector pEGFP-C1-transfected cells, proteins emitted green fluorescence upon exposure to a blue laser when observed by fluorescence microscopy, whereas no fluorescence was observed in control cells.

### Detection of antibody responses in immunized mice

**Fig. 2:**
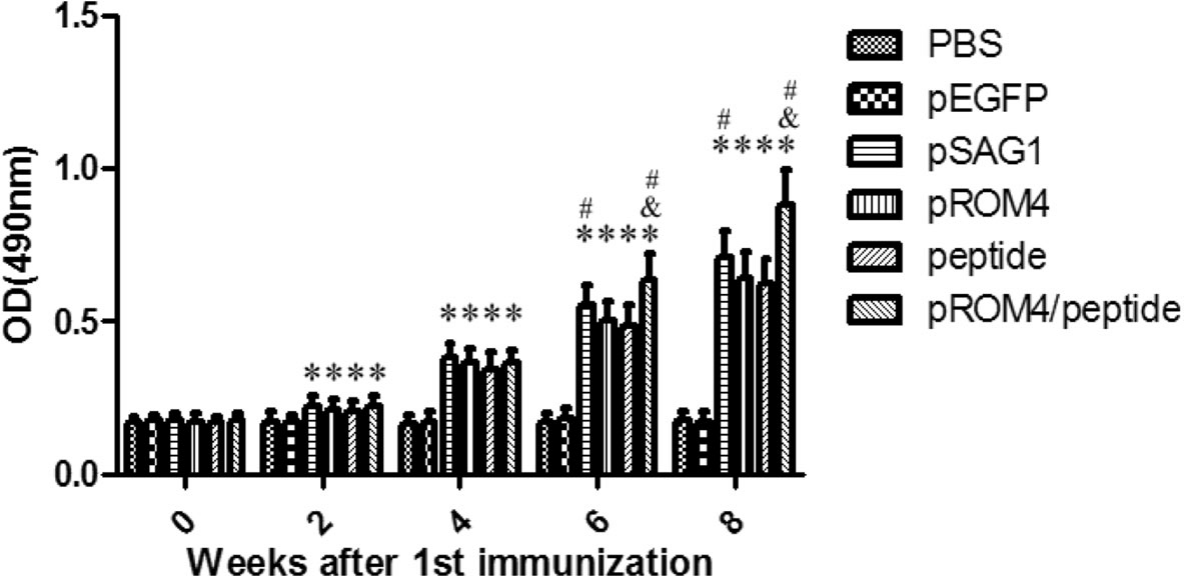
Detection of specific IgG antibodies in sera of vaccinated mice. Sera was collected 2 days before each immunization and determined using ELISA. All samples were performed four times. The results are mean of 27 mice per group and expressed as the mean of the optical density of 490 ± SD. * *P* < 0.05, as compared with PBS and pEGFP-C1; # *P* < 0.05, as compared with ROM4 or peptide; & *P* < 0.05, as compared with pSAG1

The levels of *T. gondii-*specific IgG antibodies in immunized mice were determined by ELISA at weeks 0, 2, 4, 6, and 8. Elevated IgG levels were detected in the sera of mice immunized with pSAG1, peptide, pROM4, or pROM4/peptide, compared to negative controls (PBS or pEGFP-C1) (*P* < 0.05) (Fig. 2). Importantly, *T. gondii-*specific IgG antibodies were not detected in the sera of mice injected with PBS or pEGFP-C1. Furthermore, mice immunized by pROM4/peptide generated the highest levels of *T. gondii-*specific IgG antibodies among all group (respectively increased by 24%,38%, and 41% compared with pSAG1, pROM4, or peptide immunization, *P* < 0.05). No statistical differences were found between PBS and pEGFP-C1, pROM4 and peptide (*P* > 0.05).

**Fig. 3:**
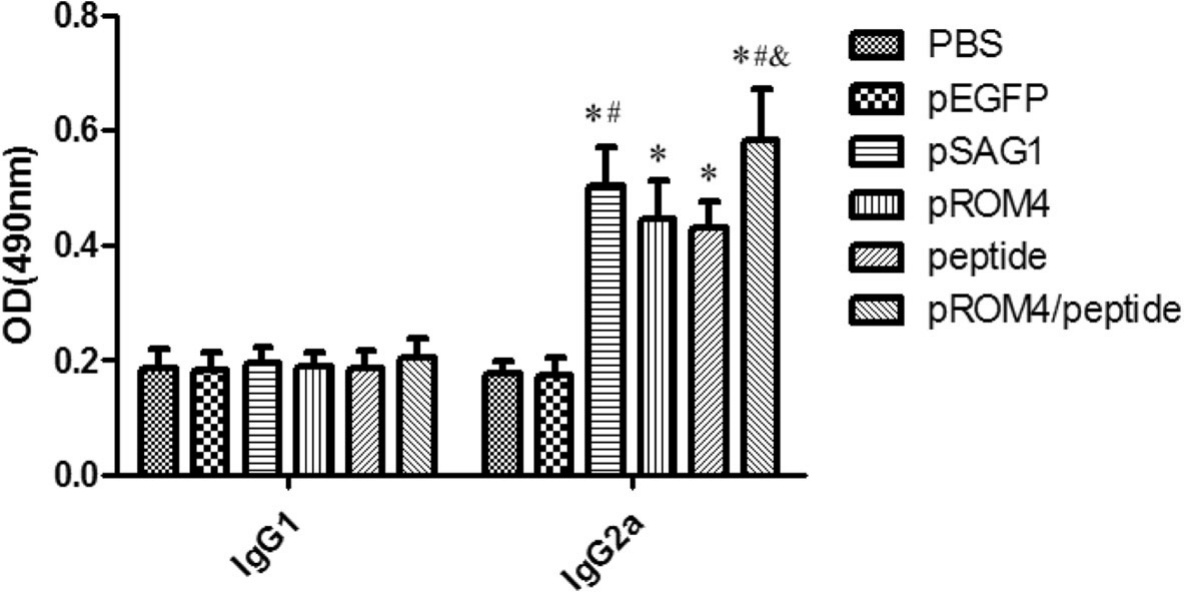
Detection of IgG1 and IgG2a levels in immunized mice sera by ELISA. Sera was collected at 2 weeks after the last injection and detected by ELISA. All samples were performed four times. The results are mean of 27 mice per group and expressed as the mean of the optical density of 490 ± SD. * *P* < 0.05, as compared with PBS and pEGFP-C1; # *P* < 0.05, as compared with ROM4 or peptide; & *P* < 0.05, as compared with pSAG1

The levels of IgG subclass (IgG1 and IgG2a) at 2 weeks after the last injection were assayed to determine whether a Th1 and/or Th2 response was elicited (Fig. 3). An apparent predominance of IgG2a over IgG1 was detected in immunized mice, suggesting a shift toward a Th1 type response. Furthermore, IgG2a levels in mice immunized with pROM4/peptide respectively increased by about 2-fold compared to negative controls and 16%, 31%, 36% compared to mice injected with pSAG1, pROM4 or peptide (*P* < 0.05). IgG2a levels in mice immunized with pSAG1 were increased by 10.3% compared with mice injected with pROM4, but with no statistical difference (*P* > 0.05). There was no statistical difference in IgG2a levels between the pROM4 and peptide groups (*P* > 0.05).

### Cytokine production

Culture supernatants of immunized splenocytes were obtained 2 weeks after the last injection and IFN-γ, IL-2, IL-12(p70), IL-4 and IL-10 activity determined. IFN-γ levels in mice immunized with pSAG1, pROM4, peptide or pROM4/peptide were increased about 12-fold, 10-fold, 9-fold, and 15-fold (*P* < 0.05), respectively, compared to PBS injected groups (Table 2). Moreover, mice immunized with pROM4/peptide generated higher IFN-γ levels than mice injected with pSAG1, peptide or pROM4 (*P* < 0.05). IFN-γ levels in mice immunized with pSAG1 were increased by about 15% compared with pROM4, but there was no statistical difference. No difference in IFN-γ levels was found between the PBS and pEGFP-C1 groups. Significantly higher levels of IL-2 and IL-12(p70) were generated from mice vaccinated with pSAG1, peptide, pROM4 or pROM4/peptide, compared to mice immunized with PBS or an empty plasmid (*P* < 0.05). IL-2 levels in pROM4/peptide immunized mice increased by 38%, 62%, and 63%, compared to pSAG1, pROM4 or peptide groups (*P* < 0.05), respectively, and the highest IL-12 level also was detected in the pROM4/peptide group (*P* < 0.05) (Table 2). The highest level of IL-4 or IL-10 was detected in mice immunized with pROM4/peptide, but there was no statistically significant difference between pROM4/peptide and other groups (*P* > 0.05).

**Table 2:** Cytokine production by splenocyte^a^ cultures from immunized BALB/c mice

Group	Cytokine production (pg/mL) ^b^		
	IFN-γ	IL-2	IL-12
PBS	48.9 ± 6.5	33.2 ± 3.56	37.5 ± 7.3
pEGFP-C1	51.1 ± 7.1	33.9 ± 5.3	38.1 ± 6.0
pSAG1	613.9 ± 59.3^*^	247.9 ± 29.0^*^	160.2 ± 35.5^*^
pROM4	531.8 ± 63.3^*^	212.4 ± 22.4^*^	146.1 ± 31.1^*^
peptide	488.6 ± 79.1^*^	210.1 ± 20.7^*^	132.9 ± 26.0^*^
pROM4/peptide	778.2 ± 93.2^*#^^&^	343.2 ± 22.4^*#^^&^	247.1 ± 48.8^*#^^&^
Group	Cytokine production (pg/mL) ^b^		
	IL-4	IL-10	
PBS	38.9 ± 8.9	39.5 ± 8.5	
pEGFP-C1	38.6 ± 10.7	41.5 ± 6.8	
pSAG1	36.5 ± 4.0	39.7 ± 5.9	
pROM4	40.2 ± 5.8	40.7 ± 4.8	
peptide	38.1 ± 7.7	37.6 ± 8.6	
pROM4/peptide	41.2 ± 5.7	42.4 ± 6.5	

^a^ Splenocytes from seven mice per group 2 weeks after the final immunization. All samples were performed four times

^b^ Values for IL-12 (p70) and IFN-γ at 96 h, IL-2 and IL-4 at 24 h, IL-10 at 72 h are expressed as mean ± SD

^*^ *P* < 0.05, as compared with PBS and pEGFP-C1; ^#^ *P* < 0.05, as compared with pROM4 or peptide; ^&^ *P* < 0.05, as compared with pSAG1

### Protection of DNA vaccine against *T. gondii*

**Fig. 4:**
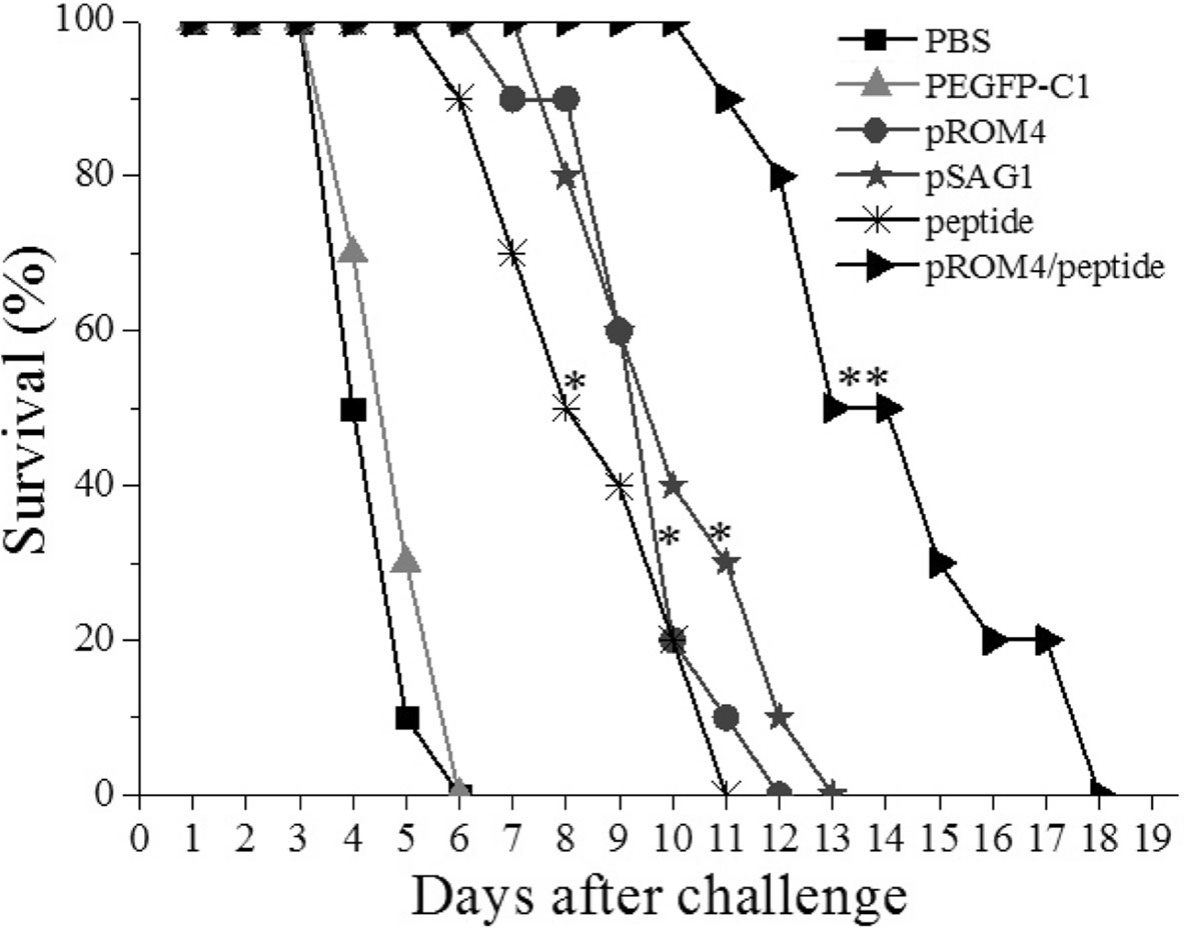
Survival curves of injected BALB/c mice against *T. gondii* challenge. The six groups of mice were challenged with 1 × 10^4^ tachyzoites of virulent *T. gondii* RH strain 2 weeks after the last immunization. Each group was composed of ten mice and survival time was monitored daily for 18 days after challenge. * *P* < 0.05, as compared with PBS or pEGFP-C1; ** *P* < 0.05, as compared with pSAG1, pROM4 or peptide

Survival days after challenge by 1 × 10^4^ tachyzoites of *T. gondii* RH strain were tabulated (Fig. 4). Mice injected with PBS or empty vector developed some symptoms characterized by reduced feeding, arched back, vertical hair and feces around the anus beginning at 2 days. Mice also started to die at 3 days after tachyzoite challenge, and all mice in negative controls were dead at day 6 postinfection. Mice showed symptoms at later timepoints and survived longer following single or multiple-immunization compared with the PBS or pEGFP-C1 groups (*P* < 0.05). Moreover, mice immunized with pROM4/peptide exhibited the latest onset of symptoms (beginning at 9 days after challenge) and the longest survival time (18 days) among all groups (*P* < 0.05).

Two weeks after the last injection, all the mice were challenged intragastrically with 20 cysts from the *T. gondii* PUR strain to evaluate the protective effect of the DNA vaccine. Brain cysts were reduced in mice vaccinated with pSAG1, pROM4, peptide, or pROM4/peptide compared to mice injected with PBS or pEGFP-C1 (*P* < 0.05) (Table 3). The cyst numbers were reduced to 57% of controls in the brains of mice immunized with pROM4/peptide. However, no statistically significant difference was found between the pSAG1, pROM4 and peptide groups (*P* > 0.05), or between the PBS and pEGFP-C1 groups (*P* > 0.05).

**Table 3:** Brain cyst burden in injected mice after infection with cyst of PRU strain

Challenged group^a^	Brain cysts per mouse (mean ± SD) ^b^
PBS	1283 ± 193
pEGFP-C1	1251 ± 199
pSAG1	812 ± 92^*^
pROM4	761 ± 102^*^
peptide	827 ± 100^*^
pROM4/peptide	551 ± 89^*#^^&^

^a^ Ten mice from each group were challenged intragastrically by 20 cysts 2 weeks after the last immunization

^b^ The mean number of cysts of each group was obtained from every mice brain cysts in the group

* *P* < 0.05, as compared with PBS or pEGFP-C1; ^#^ *P* < 0.05, as compared with ROM4 or peptide; ^&^ *P* < 0.05, as compared with pSAG1. All samples were performed four times

## Discussion

In the present study, ROM4 B-cell and T-cell epitopes were analyzed using DNAStar software and online services. This approach yielded several potential ROM4 T-cell epitopes, suggesting that it may serve as a vaccine against *T. gondii.* Following bioinformatics analysis, the ROM4 gene was cloned into the eukaryotic expression plasmid pEGFP-C1, and recombinant plasmids transfected into HEK 293-T cells. The results showed that the recombinant plasmid pROM4 was efficiently transcribed and expressed in eukaryotic cells.

Cellular immunity mediated by T cells plays an important role in *T. gondii* infection [29]. To develop an effective vaccine against toxoplasmosis, it is necessary to elucidate which type of Th cell-mediated immune response is elicited. Several studies have indicated that CD4+ and CD8+ T cells play a major role in the anti-*T. gondii* response [30, 31]. Cytotoxic CD8+ T cells and the Th1 cytokine IFN-γ play an important role in *T. gondii* immunity [30, 32]. CD4 + T lymphocytes are divided into two subtypes, Th1 and Th2, based on the cytokines produced post-stimulation. Th1 cells produce IL-2, IL-12 and IFN-γ, and Th2 cells secrete IL-4, IL-5, IL-6, and IL-10 [32]. IFN-γ plays a leading role in restricting both acute phase tachyzoite breeding and chronic phase bradyzoite activation in the cysts. Studies have shown that good DNA vaccines tend to stimulate a Th1-type rather than a Th2-type immune response [8, 33]. The Th2 cytokines IL-4 and IL-10 limit the differentiation of CD4+ T cell to Th1, thereby decreasing the level of IFN-γ [34, 35]. In addition, B lymphocytes play an important role in anti-infection immunity by producing IgG anti-*T. gondii* antibodies, and inhibiting parasite and host cell adhesion, especially during vaccine-induced protective immunity [36, 37].

In the current study, IgG antibody levels in immunized mice were significantly higher than in negative controls, illustrating that the vaccine constructed here induces a sufficient amount of *T. gondii*-specific IgG antibodies. IgG, IgG2a, IL-2, IL-12 and IFN-γ levels in immunized mice were higher than in negative controls, and IgG1, IL-4, and IL-10 levels were similar between all groups. These results suggest that pSAG1, pROM4 and peptide mainly induced a Th1 immune response. In addition, IgG and IgG2a levels in pSAG1 immunized mice were higher than in pROM4 immunized mice. This is not in accord with the results of the bioinformatics analysis, suggesting that bioinformatics analysis is predictive and that the predicted protein antigenic epitopes need to be verified by an experimental approach.

To assess the protective efficacy of the vaccine, BALB/c mice were infected with *T. gondii* tachyzoites and cysts. The results showed that the onset of symptoms were later in immunized mice, which had prolonged survival times and reduced brain cysts compared with negative controls. Zhang et al. [38] constructed a pVAX-TgROM4 DNA vaccine and evaluated the immunogenicity in Kunming mice. They showed that the TgROM4 DNA vaccine induced strong humoral and cellular responses and was a potential vaccine candidate against toxoplasmosis. Our study confirmed the protective immunity of ROM4 DNA vaccine against toxoplasmosis in BALB/c mice. However, we added the ROM4/peptide group and showed that the group had the greatest protective effect. Of note, some parameters (the parasite strain, vaccine constructs, dose of vaccination, mouse strain, et al.) might affect the assessment of protective immunity in different studies [6].

DNA vaccines often induce insufficient protective immunity against *T. gondii* challenge [39]. Prime-boost vaccination strategies have been used to enhance immune responses of some DNA vaccines [5, 22]. The first immunization primes the immune response and subsequent immunizations trigger the further expansion of antigen specific cells and selection of cells with high antigen avidity [40]. Synthetic MAP contains a high concentration of the relevant antigen to induce immune responses to predefined epitopes and leads to protective immunity [25, 41]. Meng et al. [5] used the DNA prime-peptide boost vaccination regimen to enhance the effectiveness of *T. gondii* DNA vaccines, which produced a stronger immune response and better protection compared to priming with polypeptide and boosting with DNA.

The prime-boost vaccination usually include two strategies: homologous boosting and heterologous boosting. Researches display that homologous boosting is relatively inefficient at boosting cellular immunity than that of heterologous boosting,. So-called heterologous boosting means that different antigen-delivery systems are used [42]. In the present study, we selected a polypeptide and immunized mice using a DNA-priming and polypeptide-boosting regimen. The results showed that the levels of IgG, IgG2a over IgG1, IL-2, IL-12 and IFN-γ in the DNA/peptide group were highest. In addition, mice treated with the DNA/peptide exhibited the longest survival time after challenge with tachyzoites and the greatest reduction in brain cysts after infection by cysts.

## Conclusion

In conclusion, *T. gondii* ROM4 is a potential DNA vaccine candidate against toxoplasmosis. A DNA prime-peptide boost regimen based on ROM4 elicited the highest level of humoral and cellular immune responses among the immunization regimens, indicating it may be a promising approach to generate an efficient protective immune response and prevent *T. gondii* infection.
